# Facial emotion-recognition deficits in patients with schizophrenia and unaffected first-degree relatives

**DOI:** 10.3389/fpsyt.2024.1373288

**Published:** 2024-04-12

**Authors:** Minjae Bae, Jihyun Cho, Seunghee Won

**Affiliations:** ^1^ Department of Psychiatry, School of Medicine, Kyungpook National University, Daegu, Republic of Korea; ^2^ Department of Psychiatry, Gyeongsan Joongang Hospital, Gyeongsan, Gyeongbuk, Republic of Korea; ^3^ Department of Psychiatry, Kyungpook National University Hospital, Daegu, Republic of Korea

**Keywords:** schizophrenia, emotion recognition, facial emotion, 1st degree relatives, high-risk group

## Abstract

**Introduction:**

This study aimed to determine trait- and state-dependent markers of schizophrenia by investigating facial emotion-recognition (FER) deficits in remitted patients with schizophrenia and their first-degree relatives (FR).

**Methods:**

Three groups were included: the Schizophrenia group (*n*=66), their unaffected FR group (*n*=40), and healthy controls (*n*=50) who were matched for age, sex, and years of education. A facial-labeling task was used to examine FER deficits using the following eight standardized expressions: happy, fearful, disgusted, angry, sad, contemptuous, surprised, and neutral.

**Results:**

There was a poorer accuracy in the recognition of sadness and anger in the Schizophrenia group as well as in contempt in both the Schizophrenia and FR groups compared with healthy controls. The response times for the recognition of contempt, sadness, and neutral emotion were delayed in the Schizophrenia group and those for fear were delayed in the Schizophrenia and FR groups compared with healthy controls.

**Conclusion:**

Concerning the accuracy in FER, sadness and anger can be considered state-dependent markers of remitted schizophrenia, and contempt is a trait-dependent marker of schizophrenia. Similarly, for response times in FER, contempt, sadness, and neutral emotion can be considered state-dependent markers of remitted schizophrenia, while fear is considered a trait-dependent marker of schizophrenia. These findings may contribute to the early diagnosis of schizophrenia and the development of relevant therapeutic interventions.

## 1 Introduction

Among the various symptoms of schizophrenia, impairment of social function has garnered considerable interest (1), particularly concerning difficulties in interpreting social signals and recognizing other aspects of the social environment. This may be partly related to cognitive impairment in facial recognition (2). Communication of emotions through facial expressions is closely related to practical functional outcomes that are essential to social communication (3). Facial expressions are responses to internal and external stimuli caused by complex neural networks in the brain (4) and represent an immediate, observable, easy-to-evaluate, and inexpensive biomarker for brain disorders, particularly social communication disorders. Facial emotion (or expression) recognition (FER) is a domain of affective cognition impaired across various psychiatric conditions, including schizophrenia, bipolar disorder, major depressive disorder, autism, post-traumatic stress disorder, attention deficient hyperactive disorder, borderline personality disorder, etc (5). A systematic review and meta-analysis revealed differences in accuracy in the identification of each type of emotion during a FER task in several psychiatric disorders and showed that FER is a potential integrative instrument for guiding diagnosis by enabling discrimination between schizophrenia, bipolar disorder, and major depressive disorder (6).

FER deficits have been consistently reported in patients with schizophrenia. In particular, these patients show significant deficits in recognizing negative emotions (anger, fear, and sadness) compared to controls (7, 8). These relative deficits are present in the prodromal phase of psychosis (9), during the first episode of schizophrenia (10, 11), and under chronic schizophrenic conditions (12).

Interestingly, this affect-recognition deficit has also been observed in individuals with a high familial risk of developing schizophrenia (13). Studies on FER deficits among first-degree relatives of patients with schizophrenia (FR) have found significant impairment in recognizing negative emotions, particularly fear (14, 15). Other studies have shown that the ability to recognize negative emotions such as disgust and neutral emotions (16) or disgust and anger (17) was impaired in FR compared to healthy controls (HC). Furthermore, a reduced ability to recognize neutral emotions and higher accuracy in identifying fearful emotions were predictors of the transition to a psychotic disorder in ultra-high-risk participants (18). Pena-Garijo et al. (19) reported the results of an interesting study in which FER was conducted on HC who were at low or high risk for psychosis, a group with first-episode psychosis, and a group with multi-episode schizophrenia spectrum disorders. They reported that FER is early impaired in high-risk individuals and increases along the psychosis continuum. Similarly, fear recognition is impaired throughout the illness period, suggesting a possible vulnerability marker. Recently, the European Network of National Networks Studying Gene–Environment Interactions in Schizophrenia (EU-GEI) (20) published a report indicating that FER is probably an intermediate phenotype of psychosis. In South Korea, Kang et al. (21) reported that FR have FER deficits for negative emotions, such as fear and sadness.

Conversely, other reports have revealed no deficits in FER ability in families with a genetic burden of schizophrenia or psychosis. Siblings of patients with schizophrenia demonstrated similar accuracy and speed in all FER tasks compared to HC (22). Addington et al. (23) conducted longitudinal studies in high-risk clinical groups. They reported no differences in facial affect-recognition tasks between patients who did and did not convert to psychosis. The authors concluded that poorer affect recognition may be associated with vulnerability to psychosis; however, it may not be a marker for the development of psychotic illness. Park (24) conducted a FER-processing study on HC and FR with a high genetic burden of schizophrenia, using functional magnetic resonance imaging. The study reported that when performing affect-recognition tasks for fear and neutral emotion, relatives displayed abnormal brain activity in the occipito-temporo-limbic-frontal network, which is involved in FER processing. However, the two groups had no differences in the behavioral results. Based on these findings, FER deficits may either be present primarily during psychosis (i.e., state-dependent), form an integral part of the disorder (i.e., trait-dependent), or be a combination of the two (i.e., state- and trait-dependent). Trait markers represent the characteristics of biological processes that act as antecedents and play a causative role in the pathophysiology of mental illness. Trait-dependent markers are most useful when presented in clinically unaffected FR (co-familial traits) and are not limited to those who co-segregate with psychosis (25).

The heterogeneous findings regarding FER in schizophrenia may reflect the complexity of the disorder, the diversity of the participants’ characteristics, and the research methods used. To perform a well-designed FER test, several points must be considered. First, there is a need to evaluate a variety of emotions. Early studies evaluated only three emotion categories (positive, neutral, and negative) by assessing various standardized emotions, such as Ekman’s six basic emotions (sadness, happiness, surprise, anger, disgust, and fear); these were used to identify emotion-specific characteristics (26). Second, several studies have shown that poor emotional performance in patients with schizophrenia is associated with more severe symptoms (27–29). Our previous study (30) reported that the degree of the deficiency in emotional perception in patients with early schizophrenia varies depending on the severity of psychotic symptoms. Therefore, it is necessary to control the severity of psychotic symptoms. Third, mood states lead to a bias in emotional recognition (31, 32). Other conditions, such as mood symptoms or mood disorders, are often observed in patients with schizophrenia and must be controlled. Fourth, accuracy and response time are two important measures for cognitive test evaluations, including FER tasks. Most emotion-recognition studies have evaluated only the hit rate of correct responses; however, it is also necessary to evaluate the response time in patients with schizophrenia who can cooperate with performing the tests. Fifth, if patients can recognize faces of their race better than those of other races, it may be meaningful to evaluate FER tasks using faces of their race and individuals from the same country (33). Furthermore, previous studies did not use appropriate expressions to deal with various emotions or consider cultural backgrounds, such as the participant’s race. Considering these factors, FER tests using standardized facial photographs of individuals of the same cultural backgrounds or races as the participants may be more useful.

This study aimed to clarify the state- and trait-dependent markers of remitted schizophrenia by investigating FER deficits in remitted patients with schizophrenia, FR, and HC. Specifically, this study evaluated the accuracy and response times for recognizing standardized Korean facial expressions depicting eight types of emotions after controlling for several factors that can affect emotion perception.

## 2 Materials and methods

### 2.1 Participants

The study was conducted between July 2014 and December 2019. It included patients with schizophrenia (Schizophrenia group) and FR groups recruited from among the outpatients in the psychiatric department of Kyungpook National University Hospital. Patients with schizophrenia and their primary relatives volunteered for the study after learning about it through the hospital advertisement.

The diagnosis of schizophrenia for patients and probands was based on the Diagnostic and Statistical Manual of Mental Disorders (DSM-IV) (34) after a review of medical records and interviews with two psychiatrists.

Psychotic symptoms can potentially impair performance on the FER test, ultimately undermining test reliability. Correct response times cannot be calculated when the accuracy value is zero, making patient compliance a critical factor in evaluating response times. To ensure stable test cooperation, we specifically recruited patients in remission from the Schizophrenia group. The remission status is defined as follows: 1) Brief Psychiatric Rating Scale (BPRS) (35) scores of ≤3 points concurrently on each of seven BPRS items (grandiosity, suspiciousness, unusual thought content, hallucinatory behavior, conceptual disorganization, mannerisms/posturing, and blunted affect); 2) Scale for the Assessment of Negative Symptoms (SANS) (36, 37) scores of ≤2 points concurrently on the global rating items of the four domains of SANS (affective flattening, avolition-apathy, anhedonia-asociality, alogia); 3) maintenance over 6 months of simultaneous ratings of mild or less on all items is required. 4) the aforementioned symptom severity must be maintained for a minimum of 6 months (38). However, in this study, the maintenance of remission at 6 months was determined by two psychiatrists assessing the severity of symptoms through retrospective medical record review and interviews with patients and caregivers. Patients had experienced no changes in antipsychotic medication dosage for at least the last 2 months.

The relatives were first-degree biological relatives of probands without a personal history of psychiatric disorder. 90% of the participants were the proband’s siblings (age range: 18 to 49 years), and 10% were children (age range: 20 to 28 years). A complete family history of the first-degree relatives was obtained from each proband and at least one other first-degree relative. HC without a personal and family history of DSM-IV axis I or II disorders were recruited through a local advertisement. The Korean version of the Structured Clinical Interview for DSM-IV Axis I Disorders (SCID-I) (39) was administered to all participants to confirm their diagnostic eligibility.

All participants had to be aged between 18 and 50 years. To minimize natural cognitive decline or physical conditions that may affect cognitive function (e.g., menopause), the age was set to 50 years.

For inclusion in the study, all participants had to be euthymic, as evaluated by the Korean version of the Montgomery–Åsberg Depression Rating Scale (K-MADRS) (40) (clinical cut-off level: ≤8) and the Korean version of the Young Mania Rating Scale (YMRS-K) (41) (clinical cut-off level: ≤6) and not be psychotic, as evaluated by the BPRS (clinical cut-off level: ≤30). Additional exclusion criteria for the participants included head trauma, neurologic disorders, a history of alcohol or drug abuse within the previous year, mental retardation (intelligence quotient <70), physical illness that may affect cognitive function, and serious medical conditions.

All participants, including the primary relatives, are South Korean and are of the same ethnicity. All patients with schizophrenia underwent usual outpatient treatment and did not participate in formal group therapy or individual psychotherapy. Patients were prescribed second-generation antipsychotics (either one or two drugs, excluding clozapine), benzodiazepines, anticholinergics, β-blockers, and antidepressants, and they were not requested to discontinue their medications for the study. No participants continued their medications for physical illnesses.

#### 2.1.1 Ethical consideration

This study was approved by the Institutional Review Board of Kyungpook National University Hospital (KNUH-2011-01-042). All participants were briefed on the purpose and process of the study, and they provided written informed consent for participation.

### 2.2 Assessment tools

#### 2.2.1 Evaluation of clinical symptoms

In patients with schizophrenia, remission status was evaluated using BPRS and SANS. In addition, all the psychotic symptoms of the participants were assessed using BPRS, manic symptoms using YMRS-K, depressive symptoms using K-MADRS, and diagnostic eligibility using SCID-I.

##### 2.2.1.1 SCID-I

This is a semi-structured interview guide to establish DSM-IV Axis-I disorders (42). The clinician used SCID-I to confirm the diagnosis. An approved Korean version of SCID-I was used in this study (39).

##### 2.2.1.2 BPRS

This scale is based on the clinician’s interview with the patient and observations of the patient’s behavior over the previous 2–3 days (35). The patient’s family can also provide the behavior report. The BPRS consists of positivity, negativity, and affectivity subscales. It has 18 items with a 7-point scale from 1 (not present) to 7 (extremely severe). The scores range from 18 to 126, with <31, >31, >41, and >53 indicating “illness not significant,” “mildly ill,” “moderately ill,” and “markedly ill,” respectively.

##### 2.2.1.3 SANS

The 25-item SANS is used to assess negative symptoms; the scores range from 0 (no abnormality) to 5 (severe) (36, 37). This scale is divided into five symptom dimensions: affective flattening, alogia, avolition–apathy, anhedonia–asociality, and attention.

##### 2.2.1.4 K-MADRS

MADRS (43) is a 10-item, clinician-administered scale designed to measure the overall severity of depressive symptoms. Using the MADRS with a 7-point scale from 0 (not present) to 6 (extremely severe), the intensity of depressive symptoms during the past week can be measured. The Korean version of the MADRS (40) was standardized and exhibited good reliability and validity for the measurement of the severity of depressive symptoms (Cronbach’s alpha = 0.79). The total score ranges from 0 to 60, and the clinical cutoff level is 8.

##### 2.2.1.5 YMRS-K

The YMRS (44) is a clinical interview scale used to assess the severity of manic states. The scale has 11 items and is based on the patient’s subjective report of his or her clinical condition over the previous 48 hours. Additional information is based on the clinical observations made during the clinical interview. The Korean version of the YMRS (YMRS-K) (41) was standardized and exhibited good reliability and validity for the measurement of the severity of manic symptoms (Cronbach’s alpha = 0.73). The total score ranges from 0 to 60, and the clinical cutoff level is 6 or less.

#### 2.2.2 Evaluation of intelligence and motor performance

##### 2.2.2.1 Intelligence

The Korean Wechsler Adult Intelligence Scale (45) consisted of six verbal and five performance subtexts. The verbal tests were as follows: information, comprehension, arithmetic, digit span, similarities, and vocabulary. The performance subtexts were picture arrangement, picture completion, block design, object assembly, and digit symbol. Verbal IQ, performance IQ, and full-scale IQ were obtained. We administered only two subtexts (vocabulary and block design) and calculated the total IQ using an estimation method (46).

##### 2.2.2.2 Motor performance

The finger-tapping test (47) measured psychomotor speed. This test examines finger motor ability, motor concentration, and motor execution ability and measures movement speed entirely. In general, when a contralateral prefrontal disorder is present, a delay in movement speed can be observed. Using the index finger of the dominant hand, the desk is tapped as quickly as possible for 10 seconds, five times in a row. The same is done with the non-dominant hand, and the test results are compared for each hand.

#### 2.2.3 Facial emotion-recognition task

A facial-labeling task (48) was used as the FER test to examine FER deficits. This forced-choice emotion identification task displayed eight standardized facial expressions on a computer screen: happy, sad, angry, fearful, contemptuous, disgusted, surprised, and neutral emotion. Facial stimuli were valid and reliable images (the accuracy for each emotion is 0.7 or higher, the emotional intensity is 100%) obtained from the Korean Facial Expressions of Emotion database (49), with an established set of photographs based on the characteristic facial configurations by Ekman and Friesen (50, 51).

Participants were briefed on the names of the eight emotions that would be displayed. Next, they were instructed to react to the faces shown by clicking a button corresponding to the name of an emotion on the screen as quickly as possible, using a mouse. The pictures were randomly displayed in one block (16 images in total, one male and one female model for each of the eight emotions). For the practice session, one block was performed, and participants were allowed to try the same block one more time only if they did not fully understand the test process. After confirming that the participants fully understood the procedure, the actual test was conducted in four blocks (64 images in total, other models used in practice). Participants were allowed to take a short break between blocks. Before, during, and after this task, participants were instructed to return to a stable emotional state. Facial stimuli were presented for 750ms, with an interval of 4,500ms (3,000ms of reaction time and 1,500ms of feedback time). This study measured two primary measures: the accuracy of responses indicated by mean commission error rates (percentage of wrong hit responses) and response times indicated by mean correct response time for each emotion. Only responses falling within the valid response time range of 200 to 3,000ms were included in the analysis. Any correct response shown after 3,000ms elapsed was considered missing data.

### 2.3 Statistical analyses

Categorical data are expressed as frequency counts, and continuous data are expressed as means and standard deviations. Regarding the analysis of demographic homogeneity between the groups, the chi-square test was used for sex ratio analysis, and the analysis of variance (ANOVA) test was used for age and level of education analysis. When comparing the three groups in the analysis of psychopathologies, accuracy, and response times, employing a non-parametric method is appropriate because most outcome variables did not follow a normal distribution. However, Analysis of covariance (ANCOVA) was used since the three groups needed to be compared after controlling for YMRS-K and K-MADRS. Since it was confirmed that the significant results of the ANOVA and Kruskal-Wallis tests did not differ, we accepted the results of ANCOVA as valid to some extent.

In cases of significance in the ANOVA, the *post hoc* comparisons were analyzed using Tukey–Kramer’s method, and in cases of significance in the ANCOVA, the *post hoc* comparisons were analyzed using Bonferroni method. We calculated the effect size for each emotion. The effect sizes (Cohen’s *d*) were calculated based on the average standard deviation of the two means. Values of 0.2, 0.5, and 0.8 indicated small, medium, and large effect sizes, respectively (52). All statistical analyses were performed using IBM SPS Statistics for Windows (version 25.0; IBM Corp., Armonk, NY, USA), and a *P-*value <0.05 was considered significant.

## 3 Results

### 3.1 Information on participant recruitment and screening

After meeting the inclusion and exclusion criteria in the dataset and matching the three groups for age, gender, and years of education, 79 participants were allocated to the Schizophrenia group, 46 to the FR group, and 53 to the HC group.

When the accuracy of a participant’s response to a specific emotion is zero, it is impossible to accurately calculate the response time for that emotion. This can significantly impact the comparison of the response times for the eight emotions, leading to distorted results. To ensure accurate calculation of the mean response times, participants with zero accuracy in any of the eight emotions were excluded from the analysis. A total of 13 (16.5%), 6 (13.0%), and 3 (5.7%) participants from the Schizophrenia, FR, and HC groups, respectively, were excluded from the data analysis due to the inability to measure any of the eight emotions; no significant difference was observed between the groups (*P* = 0.178). Among the emotions excluded because of having zero accuracy, fear had the largest number of emotions in the Schizophrenia, FR, and HC groups, with 8, 5, and 3 participants, respectively, and exhibited no statistical significance (*P* = 0.594). The rest of the excluded emotions were out of the measurement range for one to two participants. In the final analysis, 66, 40, and 50 participants from the Schizophrenia, FR, and HC groups, respectively, were included for accuracy and response times on the FER tests.

### 3.2 Demographic characteristics, psychopathology, and neurocognitive function tests

**Table 1** shows the results of the demographic characteristics and neurocognitive function tests for the three groups. There were no significant differences among the three groups in terms of sex (*χ^2^ = *1.41, *P*=0.49), mean age (F=0.14, *P*=0.87), and duration of education (*F*=0.97, *P*= 0.38). The mean scores of BPRS (F=20.81, *P*<0.01) were significantly higher in the Schizophrenia group (24.80 ± 5.75 points) than those in the other groups, and YMRS-K (*F*=3.43, *P*=0.04) and K-MADRS (*F*=6.36, *P*<0.01) mean scores were significantly higher in the Schizophrenia group (YMRS-K: 1.05 ± 1.68; K-MADRS: 3.30 ± 3.00 points) compared with those in HC group (YMRS-K: 0.40 ± 0.78; K-MADRS: 1.52 ± 2.10 points). Notably, all three indicators’ values lay within the significant clinical cut-off levels. The overall intelligence score on the neurocognitive function tests was significantly lower in the Schizophrenia group (108.0 ± 15.4 points) than that in HC group (115.8 ± 11.8 points) (*F*=4.79, *P=*0.01). No significant differences were observed between the three groups for the evaluation of psychomotor speed, as measured by the finger-tapping test; this was true for both dominant (*F*=1.16, *P*=0.32) and non-dominant hands (*F*=0.97, *P*=0.38).

**Table 1:** Demographic data and clinical characteristics of the participants.

		Schizophrenia group (*n*=66)	FR group (*n*=40)	HC group (*n*=50)	*F* or χ	*P*	post hoc*
Sex (men/women)		29/37	15/25	25/25	1.41	0.494	
Age (years)		30.1 ± 7.1	29.4 ± 8.1	29.6 ± 5.3	0.14	0.865	
Duration of education (years)		14.3 ± 2.0	14.3 ± 1.8	14.7 ± 1.7	0.97	0.383	
Onset age (years)		23.8 ± 5.5					
Duration of illness (years)		6.35 ± 6.00					
Number of admissions		1.08 ± 1.11					
Medications							
2^nd^ generation antipsychotics, n (%)		66 (100.0)					
Monotherapy, n (%)		58 (87.9)					
Combined therapy, n (%)		8 (12.1)					
CPZ equivalents^†^, mg		322.9 ± 225.2					
Benzodiazepines, n (%)		31 (47.0)					
Anticholinergics, n (%)		41 (62.1)					
ß-blocker, n (%)		17 (25.8)					
Antidepressants, n (%)		6 (9.0)					
Psychopathology							
BPRS		24.80 ± 5.75	20.40 ± 4.61	19.66 ± 2.52	20.81	**<0.001**	1>2,3
YMRS-K		1.05 ± 1.68	0.70 ± 1.18	0.40 ± 0.78	3.43	**0.035**	1>3
K-MADRS		3.30 ± 3.00	2.50 ± 2.71	1.52 ± 2.10	6.36	**0.002**	1>3
Neurocognition							
IQ		108.0 ± 15.4	112.6 ± 12.9	115.8 ± 11.8	4.79	**0.010**	1<3
Finger-tapping test							
Dominant hand		65.6 ± 15.0	69.2 ± 10.5	68.0 ± 10.5	1.16	0.315	
Non-dominant hand		66.8 ± 15.5	70.6 ± 11.5	68.5 ± 11.8	0.97	0.380	

FR, first-degree relatives of patients with schizophrenia; HC, healthy controls; BPRS, Brief Psychiatric Rating Scale; YMRS-K, Young Mania Rating Scale (Korean version); K-MADRS, Montgomery–Åsberg Depression Rating Scale (Korean version); IQ, intelligence quotient. ^†^Dosage equivalents of chlorpromazine. Values are presented as means ± standard deviations. Analysis of variance (ANOVA) was used. *Using Tukey–Kramer’s method. The significant values are shown in bold.

### 3.3 Accuracy

**Table 2** lists the mean commission error rates for each emotion. Compared to HC group, the Schizophrenia group showed significantly higher error rates for the recognition of sadness (*F*=11.35, *P*<0.01) and anger (*F*=5.69, *P*<0.01). The Schizophrenia and FR groups had a significantly higher error rate in recognition of contempt than the HC group (*F*=7.88, *P<*0.01). No significant differences were observed in the error rates for the recognition of happiness, fear, disgust, surprise, and neutral emotion among the three groups.

**Table 2:** Mean commission error rates by participant and emotion type in the facial emotion-recognition test.

Emotion depicted in the facial stimuli	Schizophrenia group (*n*=66)	FR group (*n*=40)	HC group (*n=*50)	*F*	*P*	post hoc*
Happiness	0.021 ± 0.060	0.009 ± 0.044	0.018 ± 0.057	0.64	0.531	
Sadness	0.320 ± 0.225	0.182 ± 0.140	0.170 ± 0.152	11.35	**<0.001**	1>2,3
Anger	0.379 ± 0.251	0.267 ± 0.199	0.251 ± 0.190	5.69	**0.004**	1>2,3
Fear	0.605 ± 0.208	0.512 ± 0.265	0.504 ± 0.222	2.81	0.064	
Contempt	0.219 ± 0.263	0.162 ± 0.172	0.040 ± 0.074	7.88	**0.001**	1,2>3
Disgust	0.478 ± 0.231	0.485 ± 0.230	0.437 ± 0.250	0.92	0.401	
Surprise	0.055 ± 0.105	0.063 ± 0.098	0.082 ± 0.120	1.30	0.276	
Neutral	0.047 ± 0.087	0.037 ± 0.090	0.015 ± 0.048	1.80	0.168	

FR group, first-degree relatives of patients with schizophrenia; HC, healthy controls. Values are presented as means ± standard deviations. Analysis of covariance (ANCOVA) was performed to control the values of the YMRS-K (Young Mania Rating Scale [Korean version]) and K-MADRS (Montgomery–Åsberg Depression Rating Scale [Korean version]). *Using Tukey–Kramer’s method. The significant values are shown in bold.

**Figure 1:**
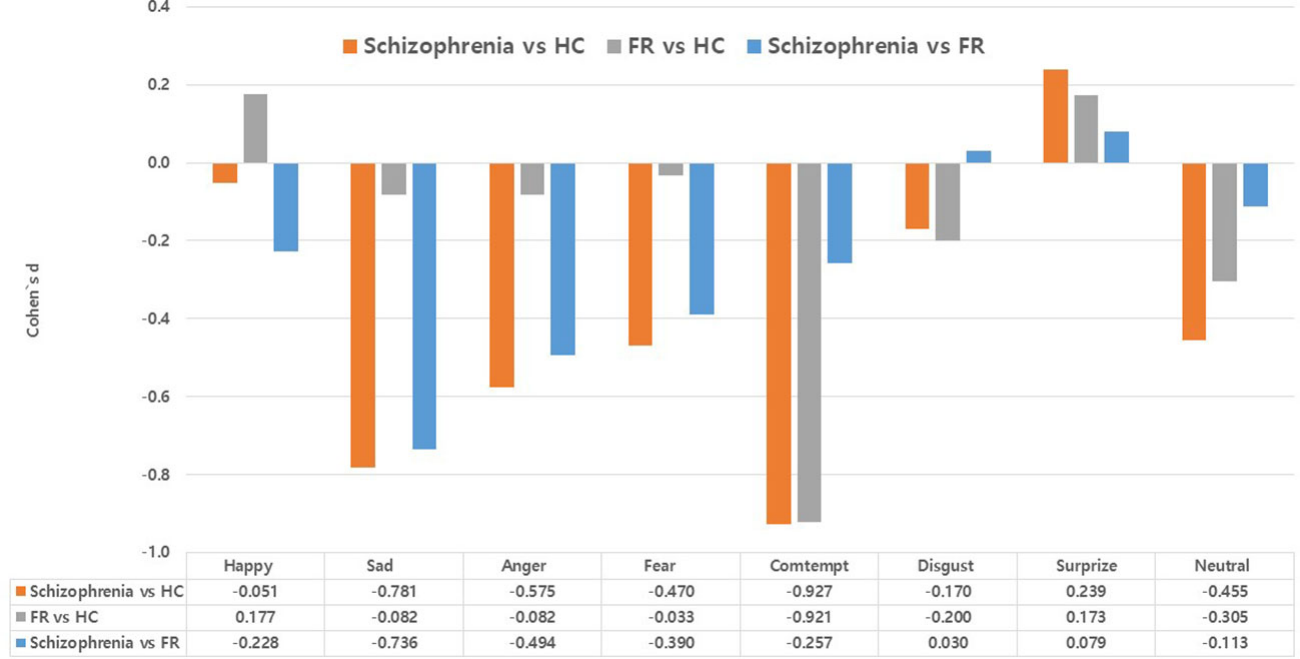
Effect size (Cohen’s d) of accuracy (commission error rates) in the facial emotion-recognition test: comparison between the Schizophrenia and healthy control (HC) groups, the first-degree relatives of patients with schizophrenia (FR) and HC groups, and the comparison between the Schizophrenia and FR groups. Values are expressed as mean with standard error.

**Figure 1** summarizes the effect sizes of the commission error rates for recognizing the eight emotions in the Schizophrenia and FR groups compared with the HC group as well as in the Schizophrenia group compared with the FR group. In the comparison between the Schizophrenia and HC groups, large effect sizes were observed for the stimuli depicting contempt (*d*=-0.93*)*, medium effect sizes were noted for sadness (*d*=-0.78) and anger(*d*=-0.58), and small effect sizes were noted for fear (*d*=-0.47), and neutral emotion (*d*=-0.46). Notably, the effect size for surprise was small, which was in the opposite direction (*d*=0.24). In the FR versus HC groups, large effect sizes were observed for the stimuli depicting contempt (*d*=-0.92) whereas small effect sizes were noted for those depicting neutral emotion (*d*=-0.31) and disgust (*d*=-0.20). In the Schizophrenia versus FR groups, medium effect sizes were observed for the stimuli depicting sadness (*d*=-0.74), whereas small effect sizes were noted for anger (*d*=-0.49), fear (*d*=-0.39), contempt (*d*=-0.26), and happiness (*d*=-0.23).

### 3.4 Response times

No significant differences were observed in the correct mean response times for the recognition of stimuli depicting happiness, anger, and disgust among the three groups. The correct response times were significantly delayed for stimuli depicting sadness *(F*=3.42, *P*=0.04) and contempt (*F*=3.83, *P=*0.02) in the Schizophrenia group compared to the HC group. The Schizophrenia and FR groups showed significantly delayed correct response times for stimuli depicting fear (*F*=4.19, *P=*0.02) compared to HC group. Furthermore, the Schizophrenia group showed a significantly delayed correct response time for stimuli depicting surprise compared to FR group (*F*=3.10, *P*<0.05) and for stimuli depicting neutral emotion compared to other groups (*F*=6.13, *P*<0.01) (**Table 3**).

**Table 3:** Mean correct response times by participant and emotion type in the facial emotion-recognition test.

Emotion depicted in the facial stimuli	Schizophrenia group (*n*=66)	FR group (*n*=40)	HC group (*n*=50)	*F*	*P*	post hoc*
Happiness	1402 ± 259	1300 ± 248	1277 ± 201	2.96	0.055	
Sadness	1755 ± 346	1636 ± 310	1541 ± 308	3.42	**0.035**	1>3
Anger	1855 ± 513	1764 ± 409	1686 ± 314	1.35	0.264	
Fear	2037 ± 492	2039 ± 358	1783 ± 394	4.19	**0.017**	1,2>3
Contempt	1675 ± 495	1581 ± 471	1365 ± 293	3.83	**0.024**	1>3
Disgust	2156 ± 517	2006 ± 426	1928 ± 504	1.85	0.161	
Surprise	1548 ± 300	1395 ± 257	1451± 242	3.10	**0.048**	1>2
Neutral	1344 ± 236	1218 ± 190	1191 ± 171	6.13	**0.003**	1>2,3

FR group, first-degree relatives of patients with schizophrenia; HC, healthy controls. Values are presented as means ± standard deviations. Analysis of covariance (ANCOVA) was performed to control the values of the YMRS-K (Young Mania Rating Scale [Korean version]) and K-MADRS (Montgomery–Åsberg Depression Rating Scale [Korean version]). *Using Tukey–Kramer’s method. The significant values are shown in bold.

**Figure 2:**
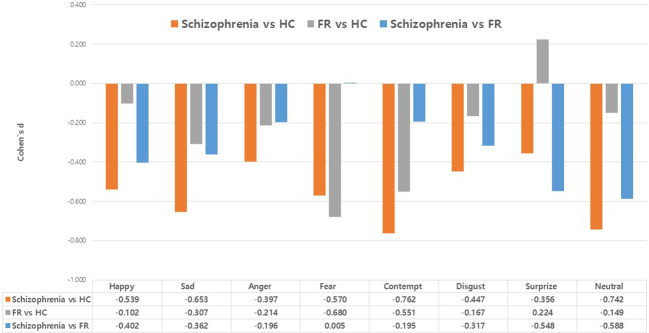
Effect size (Cohen’s d) of the response times in the facial emotion-recognition test: comparison between the Schizophrenia and healthy control (HC) groups, the first-degree relatives of patients with schizophrenia (FR) and HC groups, and the comparison between the Schizophrenia and FR groups. Values are expressed as mean with standard error.

**Figure 2** summarizes the effect sizes for the response times for eight emotions in the Schizophrenia and FR groups compared with the HC group as well as in the Schizophrenia group compared with the FR group. In the comparison between the Schizophrenia and HC groups, medium effect sizes were observed for the recognition of contempt (*d*=-0.76), neutral emotion (*d*=-0.74), sadness (*d*=-0.65), fear (*d*=-0.57), and happiness (*d*=-0.54) whereas small effect sizes were observed for the recognition of disgust (*d*=-0.45), anger (*d*=-0.40), and surprise (*d*=-0.36). In FR versus HC groups, medium effect sizes were observed for the recognition of fear (*d*=-0.68) and contempt (*d*=-0.55) whereas small effect sizes were observed for the recognition of sadness (*d*=-0.31) and anger (*d*=-0.21). The surprise was the small effect size, which was in the opposite direction (*d*=0.22). In the Schizophrenia versus FR groups, medium effect sizes were observed for the stimuli depicting neutral emotion (*d*=-0.59), and surprise (*d*=-0.55), and small effect sizes were noted for happiness (*d*=-0.40), sadness (*d*=-0.36), and disgust (*d*=-0.32).

## 4 Discussion

To identify the state and trait markers of schizophrenia, this study compared the degree and type of FER deficits in remitted schizophrenia, FR, and HC. To account for potential factors that could impact emotional recognition, we controlled for age, sex, education level, psychopathology, psychomotor speed, culture, and race. Standardized facial expressions representing eight distinct emotions were presented as facial stimuli. The facial expressions were modeled by individuals of the same culture and race as the study participants. In addition to measuring the accuracy of recognition of the individual emotions, differences between them in reaction times were also recorded.

There were no significant group differences in sex, age, or duration of education, indicating that the main variables that may affect cognitive function were well-adjusted. Patients` average duration of illness was 6.35 years, and the average number of hospitalizations was 1.08, indicating that most patients were not in a chronic state and remained stable with medication administered through the outpatient facility. Intelligence was estimated using two subtests; while it was high in all groups, it was lower in the Schizophrenia group than in HC group, in agreement with the results of other studies. The mean BPRS values were significantly higher in the Schizophrenia group than in the other groups. The YMRS-K and K-MADRS mean scores were significantly higher in the Schizophrenia group than in HC group. However, all values were within the clinical cut-off levels, indicating that the patients with schizophrenia were in remission and that psychotic and mood symptoms were stable. Thus, it can be suggested that the associated psychopathology was well-controlled. However, to minimize the effect of psychopathology on cognitive function, we treated the values of the YMRS-K and K-MADRS as covariates in the analysis of the results of the FER tasks. Furthermore, no differences in the psychomotor speed of the dominant and non-dominant hands were observed among the three groups, indicating that this parameter was also well-controlled.

With respect to the accuracy of FER, the commission error rates for stimuli depicting contempt, sadness, and anger were significantly higher in the Schizophrenia group than those in HC group; however, no differences in the rates for stimuli depicting happiness, fear, neutral emotion, surprise, and disgust were observed between the two groups. Among the three significant emotions, large effect sizes were observed for the stimuli depicting contempt, while medium effect sizes were noted for those of sadness and anger. In a comparison between the Schizophrenia and FR groups, the error rates for sadness and anger were significantly higher in the Schizophrenia group than in FR group, and both emotions showed medium effect sizes.

Most previous studies have consistently reported that patients with schizophrenia have relative deficits in their ability to recognize negative emotions (7, 8, 10, 30, 53), which is similar to our findings. Kohler et al. (27) conducted a meta-analysis and found that facial emotion recognition and discrimination abilities of patients with schizophrenia were worse than those of non-psychotic controls and were specific to negative emotions, such as fear and anger. In a meta-analysis of early schizophrenia, a large effect size was observed for the recognition of disgust, fear, and surprise, while a medium effect size was observed for the recognition of sadness and happiness. However, no differences were observed in the effect sizes for faces showing anger and neutral emotion (54). Meanwhile, Allot et al. (15) conducted an emotion-labeling task using seven emotions (excluding contempt), which is a higher number of emotions than those used in previous studies. They reported that participants with a first episode of schizophrenia performed significantly poorly in recognizing anger, disgust, and fear compared to HC. These differences in research outcomes may be attributed to methodological diversity and cultural differences. Our study is significant in that it used a total of eight emotions, including contempt, which was not evaluated in other studies, and it also controlled for several factors that could affect emotional recognition.

In this study’s analysis of FR group, the incidence of deficits in recognition of contempt in the Schizophrenia and FR groups was significantly lower than that in the HC group. In a comparison between FR and HC groups, large effect sizes were observed for the stimuli depicting contempt. As mentioned earlier, studies have investigated whether FER deficits are present in FR, and their ability to recognize different types of emotions varies across studies. Some studies have shown a deficit in recognizing fear in FR (14, 15). Others have shown differences in recognizing disgust and neutral emotion (16) and disgust and anger (17). The EU-GEI study reported that anger might serve as an intermediate phenotype for psychosis (55). A South Korean study on FR revealed a relative accuracy deficit in recognizing sadness and fear (21). These differences in the results of several studies on FR may also be attributed to variations in research methods or cultural differences. A few years ago, our research team reported differences in FER according to the severity of psychotic symptoms in patients with early-stage schizophrenia. The aforementioned study employed the same research method as the present study. The previous study showed that deficits in recognizing contempt, anger, and fear persisted in individuals regardless of the severity of psychotic symptoms, with a moderate effect size. These findings suggest that trait-dependent characteristics may exist among these emotions (30). Based on the results of the present study, for the accuracy of FER tests, sadness, and anger can be considered state-dependent markers in remitted patients with schizophrenia. In contrast, contempt can be considered a trait-dependent marker in schizophrenia.

This study found that the Schizophrenia group had significantly slower response times than HC group when presented with stimuli depicting contempt, neutral emotion, and sadness (the effect sizes of all emotions were medium) in FER. The responses to stimuli depicting fear were slower in both the Schizophrenia and FR groups than those in HC group (the effect size of fear was medium). The response time to stimuli depicting surprise was slower in the Schizophrenia group than in FR group (the effect size of surprise was medium). Most previous FER studies have focused on accuracy variables rather than response time. Further, only a few studies have focused on the response times of families with a genetic load for schizophrenia and psychosis. Reports found that the response for all emotions, regardless of the detailed emotion, was delayed in patients with schizophrenia (22) and their primary families (22, 24). Evaluating the response time is useful in several cognitive research areas, such as semantic and perceptual priming, implicit serial, sequence, and learning. Measuring the response times of correct responses by assigning a time limit may reflect real-life situations more closely (56). Many scientists seem to religiously adhere to the study of either accuracy or response time; rarely are both investigated simultaneously in a given experimental design.

Furthermore, accuracy and response time data are often critical for distinguishing between theories of cognition, and using only one of these measures may generate a skewed interpretation. Therefore, both accuracy and response time are significant values to be measured (56–58). Brain imaging studies involving patients with schizophrenia and their primary families have revealed abnormalities in processing FER information. A recent meta-analysis of existing brain imaging studies related to facial emotion processing capabilities of patients with schizophrenia revealed the following: a significant under-recruitment of the amygdala and a substantial limitation in activation throughout the ventral temporal-basal ganglia-prefrontal cortex ‘social brain’ system is responsible for difficulties faced by participants when processing facial emotion (22). Additionally, dysfunction was observed in facial expression processing in non-psychotic siblings of patients with schizophrenia similar to that of the patients, and abnormal activation was observed in both groups in the precentral and superior frontal gyri (59). This study’s results suggest that contempt, sadness, and neutral emotion are state-dependent markers for response times of FER tests in remitted patients with schizophrenia. At the same time, fear is a trait-dependent marker of schizophrenia.

This study has some limitations. First, the possibility of drug-induced cognitive impairment could not be completely ruled out, owing to the use of psychiatric medications, such as antipsychotics, benzodiazepines, and other psychotropics, in the patient group. Second, as this is a cross-sectional study, it is important to track whether these state and trait characteristics are maintained over time to investigate the recurrence of symptoms in remitted patients with schizophrenia or the onset of psychotic symptoms in FR using longitudinal follow-up studies. Third, our study lacks generalizability as it included a sample population of only one ethnicity. Consequently, FER deficits in individuals with schizophrenia from various ethnic and cultural backgrounds should be analyzed using the same research methods. Differences in FER deficits between cultures and countries have been reported in healthy individuals (60). FER deficits among patients with schizophrenia in all cultures share the same characteristics, although there are differences in FER deficits for specific emotions (61). Fourth, according to criterion (38), maintenance of a 6-month remission must be confirmed through monthly evaluations using relevant measures. However, in this study, scale evaluations were conducted once upon registration, and the maintenance of remission was clinically determined by two psychiatrists who assessed symptom severity through a retrospective review of medical records and interviews with patients and caregivers. Finally, we have a system for conducting training and evaluation using standard videos to maintain interrater reliability. However, since data were collected over a long period of time, there is a possibility of environmental or evaluator bias occurring.

Nevertheless, this study had several strengths. First, demographic variables (age, sex, and education level), psychopathology (psychotic symptoms and mood symptoms), and psychomotor ability that can affect cognitive function were controlled. Second, we used standardized facial stimuli (i.e., facial expressions of models of those of the same culture and race as that of the participants) in the study. This was considered based on the other-race effect, considering that patients with schizophrenia can recognize faces of their race better than those of other races (33). Third, this study employed eight emotions, the most examined among the studies reported. Fourth, response time, one of the major indicators of FER inadequately investigated in previous studies, was measured for each emotion. Fifth, the target patients with schizophrenia were not in a chronic disease state, did not have recent onsets of schizophrenia, and had experienced remission in terms of psychotic symptoms.

A range of phenomenological symptoms characterizes schizophrenia, and it is crucial to identify the associated risk factors for its development. Early diagnosis and therapeutic intervention in high-risk groups are among the most effective approaches for preventing and treating schizophrenia. Our findings may contribute to the early diagnosis of schizophrenia and the development of relevant therapeutic interventions. Future research is essential to determine the possibility of making novel predictions regarding the transition to schizophrenia, including factors such as neurocognitive function, which can affect FER tests.

In conclusion, our study was conducted using a facial-labeling task to compare the degree of FER deficits among patients with schizophrenia, FR, and HC and to investigate the state- and trait-dependent markers of remitted patients with schizophrenia. The Schizophrenia group showed reduced recognition accuracy for emotions of sadness and anger; both the Schizophrenia and FR groups showed reduced recognition accuracy for the emotion of contempt. Regarding the response times of FER tasks, emotions of contempt, sadness, and neutral emotion were delayed in the Schizophrenia group; and emotions of fear were delayed in the Schizophrenia and FR groups. Therefore, sadness and anger can be considered state-dependent markers for accuracy in FER in remitted patients with schizophrenia, and contempt is a trait-dependent marker of schizophrenia. Furthermore, for response times in FER, contempt, sadness, and neutral emotion can be considered state-dependent markers in remitted patients with schizophrenia, and fear as trait-dependent markers of schizophrenia. Therefore, it is necessary to consider the deficits in FER in the evaluation and follow-up of clinical progression in patients with schizophrenia and high-risk groups. In remitted schizophrenia, the deficiency in FER is a mixture of state- and trait-dependent markers, depending on the measured variable (accuracy or response time) and the type of emotion.

## Data availability statement

The raw data supporting the conclusions of this article will be made available by the authors, without undue reservation.

## Ethics statement

The studies involving humans were approved by The Institutional Review Board of Kyungpook National University Hospital. The studies were conducted in accordance with the local legislation and institutional requirements. The participants provided their written informed consent to participate in this study.

## Author contributions

MB: Visualization, Writing – review & editing, Writing – original draft, Project administration, Investigation, Formal analysis, Data curation. JC: Writing – review & editing, Software, Project administration, Investigation, Data curation. SW: Writing – review & editing, Visualization, Validation, Supervision, Software, Resources, Project administration, Methodology, Funding acquisition, Formal analysis, Data curation, Conceptualization.
